# A de novo variant of *BICRA* results in Coffin–Siris syndrome 12

**DOI:** 10.1002/mgg3.2250

**Published:** 2023-07-24

**Authors:** Youquan Tu, Chunyan Fang, Jian Xu, Yun Zhou, Mengmeng Liang, Zuozhen Yang

**Affiliations:** ^1^ Department of Pediatric Neurology Ningbo Women and Children's Hospital Ningbo China; ^2^ Department of Radiology Ningbo Women and Children's Hospital Ningbo China; ^3^ Cipher Gene LLC Beijing China

**Keywords:** *BICRA*, Coffin–Siris syndrome 12, developmental delay, stop gain, variant

## Abstract

**Background:**

*BICRA*, a transcript regulator, was identified as the genetic factor of Coffin–Siris syndrome 12 (CSS12) recently, which was characterized by diverse neurodevelopmental delays. Up to now, limited studies of *BICRA* in neurodevelopmental delay have been reported.

**Methods:**

Clinical data such as EEGs, MRIs, routine blood, and physical examination were collected. Trio whole exome sequencing (WES) of the family was performed, and all variants with a minor allele frequency (<0.01) in exon and canonical splicing sites were selected for further pathogenic evaluation. Candidate variants were validated by Sanger sequencing. *The BICRA‐related* literature was reviewed and the clinical characteristics were summarized.

**Results:**

We reported a CSS12 proband with a narrow and slightly clinical phenotype who only exhibited language developmental delay, hypotonia, and slight gastrointestinal features. WES revealed a de novo variant in exon 6 of *BICRA* [NM_015711.3: c.1666C>T, p.Gln556*]. This variant resulted in an early translation termination at 556th of BICRA, not collected in the public population database (gnomAD), and classified as pathogenic according to the ACMG guideline.

**Conclusion:**

Our results expanded the pathogenic genetic and clinical spectrum of BICRA‐related diseases.

## Graphical abstract

1. We report the language developmental delay as a new phenotype for Coffin–Siris syndrome caused by BICRA, which expands the clinical features
2. We report another nonsense variant of BICRA which expands the limited variant spectrum.

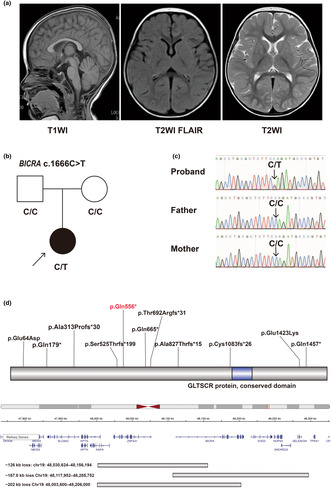

## 1 INTRODUCTION

*BICRA* was first discovered in 2000 from a 150‐kb interval of chromosome 19q13.3, this region was frequently observed in diffuse gliomas, so *BICRA* was named as Glioma Tumor Suppressor Candidate Region Gene 1 Protein (GLTSCR1; Smith et al., 2000). It was found to share high homology with *BICRAL* in the N and C terminal domain in 2018 (Alpsoy & Dykhuizen, 2018), located in the nuclei of neurons and glia of drosophila, indicating its potential role in neurodevelopment.

*BICRA* was first identified as the pathogenic gene for Coffin–Siris syndrome 12 (CSS12; MIM#619325) in nine unrelated patients (Barish et al., 2020). Seven loss of function variants, two missense variants, and other three copy number variants (CNVs) which covered *BICRA* gene fragments were discovered in these patients. Clinical features of CSS12 patients included developmental delay, intellectual disability, autism spectrum disorder, behavioral abnormalities, and dysmorphic features (Barish et al., 2020). Another nonsense case was also reported recently, which introduced a low weight, microcephaly, neurodevelopment delay, and other malfunction features boy (Asadauskaite et al., 2022). Besides, one CSS12 case with p.Ala827Thrfs*15 variant in *BICRA* was detected from a BAFopathies cohort (Chen et al., 2022).

Here, we report another girl who presented a developmental delay with a pathogenic variant which resulted in early translation termination of *BICRA*.

## 2 METHOD

### 2.1 Ethical compliance

Informed consent was obtained from her parents. This study was approved by the Institutional Review Board of Ningbo Women and Children's Hospital. Clinical characteristics, brain magnetic resonance imaging (MRI), electroencephalogram (EEG), and other examination results were collected.

### 2.2 WES and Sanger sequencing

Genomic DNA was extracted from the whole blood sample. The IDT XGen Exome Research Panel was used to capture libraries, and then, the library was sequenced on the NovaSeq 6000 Sequencing platform. Finally, clean reads at the pair ended were compared with the human reference genome (GRCh38/hg38) by the Burrows‐Wheeler Alignment tool (BWA; Li & Durbin, 2009). Variations were annotated through ANNOVAR (Wang et al., 2010), and SNPs with a minor allele frequency of <0.01 in the SNP database were obtained for further pathogenicity evaluation according to ACMG guideline (Richards et al., 2015). Sanger sequencing of candidate variants was performed on samples from the proband and her parents to validate the variation identified by WES. CNVs were analyzed by ExomeDepth, filtered by CNVs frequency database of DGV (http://dgv.tcag.ca/), annotated by disease‐associated CNVs database (ISCA, DECIPHER; Bragin et al., 2014; Miller et al., 2010), candidate CNVs were classified according to the ACMG guidelines.

### 2.3 Deleterious effect prediction

gnomAD database (http://www.gnomad‐sg.org/gene) was utilized for the probability of loss of function intolerance. The DECIPHER database (https://www.deciphergenomics.org/gene) was used to evaluate the probability of haploinsufficiency. The DOMINO database (https://www.fbm.unil.ch/domino/) was used to assess the probability that a gene harbors dominant changes.

## 3 RESULTS

### 3.1 Case presentation

This proband was born at a full turn with a normal pregnancy to nonconsanguineous healthy parents. Her parents denied her family history and genetic disorder history. She was the first child in her family. Her birth weight was 3000 g without a history of asphyxia, rescue, and other accidents. Her head circumference was within the normal range; stature was a little smaller when birth. She held her head at 6 months and started to walk at 18 months. No specific facial features or stereotypical hand movements were presented.

When she was 25 months old for the initial evaluation, she had communication difficulties with language delay, dystonia, less eating, frequent constipation, and dry stool. Her motor development was delayed, manifested as unstable walking and unable to run. Investigations of other organs (skin, heart, eye, liver, lung, kidney, etc.) revealed a normal result. Giant cell astrocytoma, cortical tubers, or subependymal nodules were also not observed.

Laboratory examination revealed that serum levels of several components were normal, including lactic acid, blood ammonia, pyruvate and β‐hydroxybutyric acid. Blood metabolic screening was normal.

**FIGURE 1:**
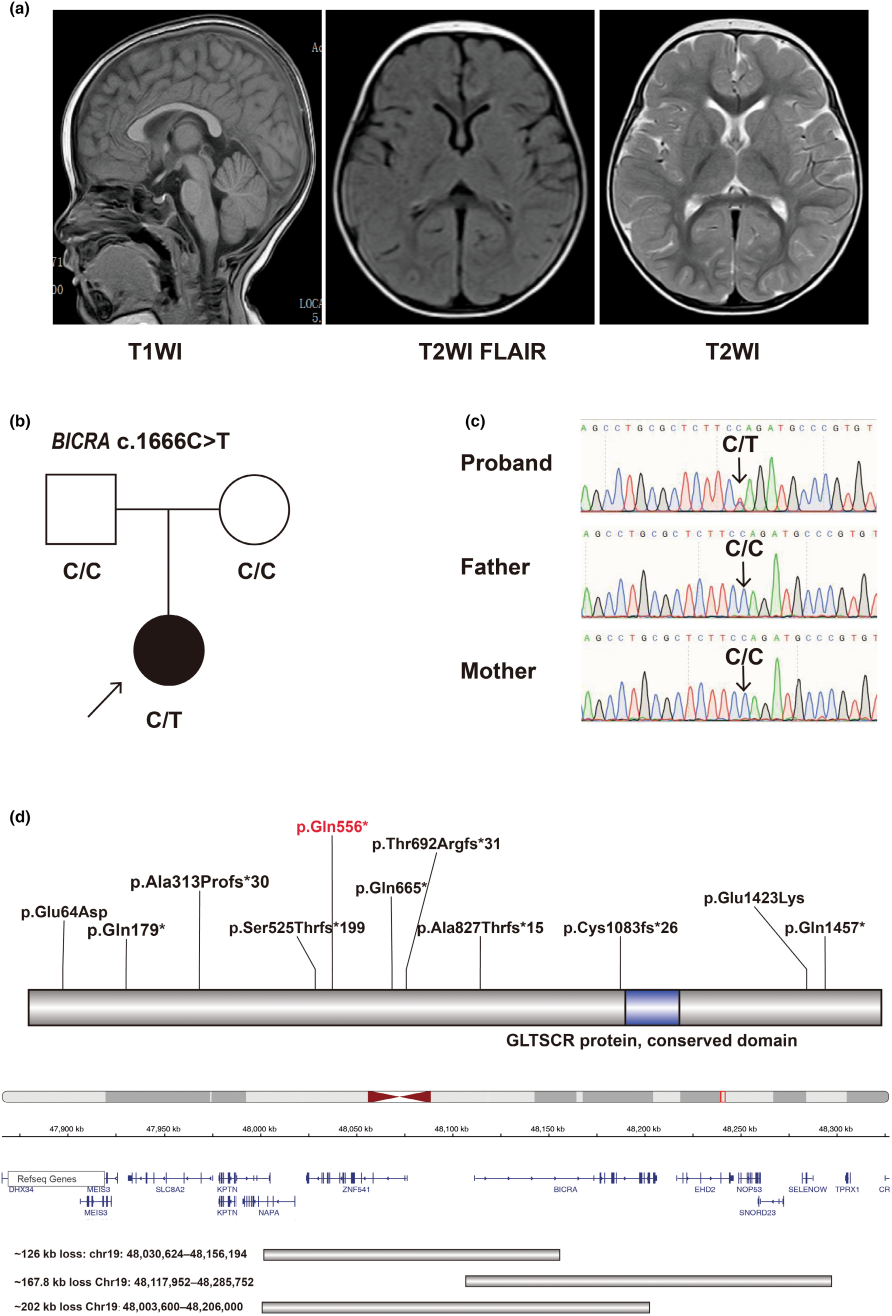
Clinical and genetic results of *BICRA*. (a) Characteristic changes in the MRI findings of the proband. Results from T1WI, T2WI, T2WI FLAIR were shown. Magnetic resonance imaging (MRI) showed a speckled signal at the left frontal lobe. (b) Pedigree chart and genotype information of the family. Black arrow: proband. (c) Genotype of the whole family, which was validated by Sanger sequencing. De novo variant was confirmed. (d) Summary of reported variants for *BICRA*. Variants written in black are reported. A variant written in red is discovered in our patient.

The EEGs did not show any abnormality, and the brain MRI showed a speckled signal at the left frontal lobe (Figure 1a).

### 3.2 Identification of de novo *BICRA* variant and literature review

A de novo variant was detected by exome sequencing from the patient: *BICRA* [NM_015711.3: c.1666C>T, p.Gln556*] (Figure 1b). The variant was confirmed by Sanger sequencing (Figure 1c). It is predicted to cause the arginine at position 556 to become a stop codon and cause premature truncation of the protein, and likely cause loss of function of the BICRA protein. This variant was not collected in the ExAC, gnomeAD, 1000genome, and ClinVar databases. No other abnormal variants such as CNVs and mitochondrial‐related variants were found. All published variants of *BICRA* were collected and displayed among the whole BIRCA protein (Figure 1d).

Combining our case and reported cases, we found the incidence of each system was quite diverse (Table 1): Neurological (14/14), Behavioral problems (6/14), Gastrointestinal (11/14), Ophthalmological (7/14), Cardiologic (5/14), Urinary (3/14), Orthopedic (6/14), Growth (11/14), Facial features (13/14), Digital anomalies (6/14), Dental anomalies (1/14) (Asadauskaite et al., 2022; Barish et al., 2020). Most reported cases exhibited intellectual disability (ID) (12/14), and some of them also exhibited other neuro‐development disorders such as autism spectrum disorder (5/14), epilepsy (2/14) (Asadauskaite et al., 2022, Barish et al., 2020). However, our case showed language developmental delay without ID or epilepsy (Table 1, Supplementary Material). Besides, 42.8% of cases showed behavioral problems (6/14) such as emotional outbursts, irritability, impulsivity, aggressiveness, or hyperactivity. These abnormal behaviors were not observed in our proband. 57.1% cases (8/14) reported feeding difficulties, our proband could eat normally, just eat less than other children (Supplementary Material).

**TABLE 1:** Clinical features summary of *BICRA‐*related disorder.

Features	Neurological	Behavioral	Gastrointestinal	Ophthalmological	Cardiologic
All cases (14)	14/14	6/14	11/14	7/14	5/14
Reported cases (13)	13/13	6/13	10/13	7/13	5/13
Our case	Yes	No	Yes	No	No

Features	Urinary	Orthopedic	Growth	Facial	Digital anomalies	Dental anomalies
All cases (14)	3/14	6/14	11/14	13/14	6/14	1/14
Reported cases (13)	3/13	6/13	10/13	13/13	6/13	1/13
Our case	No	No	Yes	No	No	No

*Note*: BICRA transcript: NM_015711.3.

### 3.3 Predicted deleterious effect of *BICRA* de novo variant

*BICRA* exhibited a high probability of loss of function (LoF) intolerance (pLI) score of 0.98 (gnomAD v2.1; Karczewski et al., 2020), classified *BICRA* as “likely dominant” in DOMINO (Quinodoz et al., 2017) database with the probability of being AD score of 0.68. Considering all reported cases also exhibited as autosomal dominant heritage model (Asadauskaite et al., 2022; Barish et al., 2020), the de novo variant, in this case, was considered as the pathogenic factor for the development delay.

## 4 DISCUSSION

*BICRA* was first cloned in 2000, and named Glioma Tumor Suppressor Candidate Region Gene 1 Protein (GLTSCR1) due to its frequent deletion in diffuse gliomas. Then its diverse expression in heart, brain, muscle, lung, liver, and kidney was detected. It was reported to activate the transcription of bromodomain protein 4 (Brd4), which played a critical role in development, cancer progression, and virus‐host pathogenesis (Rahman et al., 2011). Recently it was reported as the pathogenicity of Coffin–Siris syndrome 12 (OMIM: 619325), which was a neurodevelopmental disorder featured by the global developmental delay with variably intellectual development, language delay, or behavioral abnormalities (autism or hyperactivity).

Barish S et al. reported 12 probands with rare variants in *BICRA*. The clinical features of the probands included developmental delay, ID, autism spectrum disorder, and behavioral abnormalities, as well as dysmorphic characteristics. The lacking of fifth digit/nail hypoplasia phenotype was regarded as a hallmark of most SSRIDDs (Barish et al., 2020). Another similar two cases were also reported recently (Asadauskaite et al., 2022; Chen et al., 2022).

Here, we detected a novel heterozygous nonsense variant in *BICRA*. It was not collected in public population databases such as gnomAD and resulted in an early translation termination around the first 1/3 site of total protein, which may cause nonsense‐mediated mRNA decay for *BICRA*, and further result in LoF of *BICRA*. This supported the pathogenic mechanism of *BICRA*‐related SSRIDD. Up to now, a total of 9 LoF variants, 2 missense variants and 3 CNVs were reported (Figure 1d).

In this study, we reported a 25‐month girl, who carried a new nonsense variant in *BICRA*. Her main clinical features included language developmental delay, hypotonia, and slight gastrointestinal problems. Compared with reported cases, she did not exhibit abnormality in facial features, growth, or behavioral issues. Since her language was delayed, it was a little difficult for her to communicate and play with other children. No characteristics of autism or behavioral abnormalities were observed.

## 5 CONCLUSION

We reported a de novo *BICRA* variant that may lead to neurodevelopmental delay. To the best of our knowledge, limited studies focused on *BICRA*. We summarized the clinical characteristics of the limited patients with neurodevelopmental delay caused by the *BICRA* variant. Our report expanded the phenotype and genotype of *BICRA‐*related disorder.

## AUTHOR CONTRIBUTIONS

Youquan Tu: Conceptualization, Methodology, Supervision, Manuscript‐Reviewing; Chunyan Fang, Jian Xu: Clinical data collection. Yun Zhou: Writing‐Original draft preparation, Software; Mengmeng Liang, Zuozhen Yang: Data analysis, Investigation.

## FUNDING INFORMATION

This work was supported by the Zhejiang Medical and Health Technology Project (No.2022KY1154).

## CONFLICT OF INTEREST STATEMENT

The authors declare no conflict of interest.

## ETHICS STATEMENT

This study was approved by the Ethics Committee of West China Second University Hospital of Sichuan University. Informed consent was obtained from the proband and their families. Clinical manifestations, EEG, other clinical results, and gene variations were investigated.

## Supporting information


Data S1

## Data Availability

The datasets used and analyzed during the current study are available from the corresponding author on reasonable request.
